# Realization of affiliation goals, interpersonal identity development, and well-being: effects of the implicit affiliation motive among German and Zambian adolescents

**DOI:** 10.3389/fpsyg.2024.1355213

**Published:** 2024-06-27

**Authors:** Jan Hofer, Holger Busch, Anitha Menon

**Affiliations:** ^1^Department of Developmental Psychology, Trier University, Trier, Germany; ^2^Department of Psychology, University of Zambia, Lusaka, Zambia

**Keywords:** implicit affiliation motive, interpersonal identity development, friendship, successful realization of affiliation goals, well-being, culture

## Abstract

**Introduction:**

Across various cultural contexts, success in goal realization relates to individuals’ well-being. Moreover, commitment to and successful pursuance of goals are crucial when searching for a meaningful identity in adolescence. However, individuals’ goals differ in how much they match their implicit motive dispositions. We hypothesized that successful pursuance of affiliation goals positively relates to commitment-related dimensions of interpersonal identity development (domain: close friends) that, in turn, predict adolescents’ level of well-being. However, we further assumed that the links between goal success and identity commitment are particularly pronounced among adolescents who are characterized by a high implicit affiliation motive.

**Methods:**

To scrutinize the generalizability of the assumed relationships, data were assessed among adolescents in individualistic (Germany) and collectivistic (Zambia) cultural contexts.

**Results:**

Regardless of adolescents’ cultural background, we found that commitment-related dimensions of interpersonal identity development mediate the link between successful attainment of affiliation goals and well-being, particularly among adolescents with a pronounced implicit affiliation motive; that is, the strength of the implicit affiliation motive moderates the association between goal success and identity commitment.

**Conclusion:**

We discuss findings concerning universal effects of implicit motives on identity commitment and well-being.

## 1 Introduction

Commitment to personal meaningful goals and their successful pursuance and progress in identity development have often been found to predict individuals’ subjective well-being (Brunstein, 1993; Hofer et al., 2007). Moreover, findings indicate that particularly the pursuance of motive-congruent goals relate to progress in personality development and enhanced levels of well-being (Brunstein et al., 1998; Hofer et al., 2006). In the present study, we hypothesize that the successful realization of affiliation goals relates to interpersonal identity development, which, in turn, relates to individuals’ subjective well-being. Moreover, we hypothesize that the implicit affiliation motive moderates the association between goal realization and identity formation: their relation should increase with an increase in the implicit affiliation motive. Finally, we do not expect any variations in the moderating effect of the implicit affiliation motive across German and Zambian adolescents.^1^

### 1.1 Goals and well-being

People pursue goals from different domains of life to varying degrees. For example, some adolescents assign much importance to excelling at school and thus pursue achievement goals. In the present study, the focus is on affiliation goals, that is, goals that refer to the establishment, maintenance, and restoration of positive interpersonal relationships (e.g., Hofer and Hagemeyer, 2018). Generally, goal realization can be impeded for many reasons. Thus, people differ in their goal realization, which we understand as the individual’s evaluation of their success in their goal pursuit. This concept is related to, but differs from, goal importance (for an overview of goal dimensions, see, e.g., Emmons, 1986).

As goals provide a sense of direction and meaning (Bühler and Massarik, 1968), we expect that goal realization increases well-being. In fact, this notion is supported by meta-analysis, showing a positive association between goal realization and subjective well-being (Klug and Maier, 2015). Subjective well-being in the context of this meta-analysis was defined in terms of life satisfaction, affective well-being, and a combination of these factors. Based on this precedence, we will also use an indicator of well-being that combines cognitive (i.e., life satisfaction) and affective (i.e., positive affect, negative affect, and anxiety) components.

### 1.2 Goals, well-being, and implicit motives

Nonetheless, not all goals hold the same promise of contributing to well-being. Schultheiss et al. (2008) distinguish between a cold and a hot mode of goal pursuit. The latter describes a goal pursuit that is in line with a high corresponding implicit motive. According to McClelland et al.’s (1989) theory, motives (e.g., affiliation) are represented in two ways: The first is the explicit motive system, which subsumes conscious representations of one’s own motivational self-concepts, that is, goals, values, beliefs, and attitudes; the second is the implicit motive system, with implicit motives being affective preferences for the approach toward certain incentives (indicating opportunities for need satisfaction) and the avoidance of certain disincentives (pointing to the likelihood of need frustration).

In other words, self-attributed or explicit motives represent motivational end-states we consciously define as desirable. Thus, individuals can easily report on their goals and values. Components of the explicit motivational system are learnt through verbal instructions by parents or other significant socialization agents and predict people’s immediate behavior in situations in which they cognitively decide on a course of action in line with their self-concept (McClelland et al., 1989). In contrast, implicit motives that are primarily shaped by classical and instrumental conditioning during early development when language mastery has not yet been established energize, select, and direct an individual’s spontaneous behavior without conscious awareness. Thus, implicit motives need to be assessed through thematic apperception techniques such as the Picture Story Exercise (McClelland et al., 1989).

For instance, emotional closeness felt in interactions with other people represents an incentive for the affiliation motive; that is, the hot mode of goal pursuit is given when an individual strives to realize affiliation goals and, due to their implicit affiliation motive, can consummate the affective incentives that the goal pursuit provides. Thus, implicit motives represent a kind of weighing disposition, which determines to what extent certain strivings and activities are considered emotionally pleasant and fulfilling (Schultheiss et al., 2008). A host of studies have supported the notion that well-being benefits from an alignment of explicit goals and implicit motives (e.g., for congruence of affiliation goals and the implicit affiliation motive: Hofer and Chasiotis, 2003).

### 1.3 Goals, implicit motives, identity, and well-being

A good match of the implicit and explicit motive system seems to be beneficial not only for well-being but also for personality development. Hofer et al. (2006) provide evidence that young adults high in both implicit and explicit affiliation motives have a more mature interpersonal identity. Erikson (1966) introduced the term identity to denote the main developmental task of adolescence, which is to develop “a new sense of continuity and sameness” (p. 289) during the transition from childhood to adulthood. For example, adolescents are required to choose a career path for their adult life. In the original conceptualization, this encompasses two processes (Marcia, 1966): first, adolescents gather information on potential careers (i.e., exploration), and second, they make a decision on which specific career to pursue (i.e., commitment). Indeed, such identity choices need to be made not only for the occupational domain but also in other areas of life, such as religious and political convictions. Most relevant for the present study, however, is the interpersonal area of life, which requires identity work during adolescence, such as ideas about romantic relationships and close friendships.

More recently, some models have proposed a reconceptualization of the processes that drive identity formation (e.g., Luyckx et al., 2008). The present study follows Crocetti et al.’s (2008) three-dimensional identity model. This model also uses *commitment* to refer to the degree of identification an individual has made to some identity element (e.g., a career to pursue). It also features an exploration process, namely, *exploration in-depth*, which describes an individual’s tendency to try to learn more about the identity element to which they have committed. Finally, it introduces *reconsideration of commitment* as a process that allows for the dissolution of existing commitments: In case of dissatisfaction with their current commitments, reconsideration of commitment means that individuals explore in breadth; that is, they seek information about alternative identity commitments.

Although each of these processes is important for adolescents in achieving a satisfactory solution for the developmental task of identity formation, they do relate differently to well-being (Crocetti, 2018). While commitment relates positively to well-being, reconsideration of commitment is negatively associated with well-being because it entails insecurity about one’s present identity. How exploration in-depth and well-being correlate is less straightforward because on the one hand exploration *per se* is functional as it increases identity options, but on the other hand, exploration can become dysfunctional when adolescents cannot let go of the exploration process, thus ruminating competing identity options (Luyckx et al., 2008). Despite some variations, this pattern can be found in adolescents from various cultural contexts (Dimitrova et al., 2015).

### 1.4 The role of the cultural context

Unfortunately, most findings in psychology still stem from Western samples (Arnett, 2008; Thalmayer et al., 2021). Fortunately, cross-cultural research steadily enhances our knowledge on behavioral and psychological processes of individuals who were raised in diverse cultural contexts. There is, however, a tendency to highlight differences in psychological processes and behavior across cultural groups, rather than examining similarities (Brouwers et al., 2004; Poortinga, 2011). In our view, it is crucial to reveal similarities and determine whether basic principles in human functioning can be identified in humans universally. We accept that behavior may vary across cultural contexts due to the effects of sociocultural orientations such as behavioral norms and role obligations (Chiu and Hong, 2007), but we challenge the idea that culture-bound experiences inevitably lead to variability in basic psychological mechanisms. Thus, although cultures may differ in the degree to which they provide chances for and encourage exploration, the task of identity formation can easily be assumed to be universal. Indeed, research indicates the cross-cultural applicability of the three-process model of identity formation proposed by Crocetti et al. (2008, 2015; Dimitrova et al., 2016). Given the linkage of identity development and well-being in diverse cultural contexts (e.g., Hofer et al., 2007; Dimitrova et al., 2015; Crocetti, 2018), deepening our understanding of the identity formation processes by including personal goals and implicit motive dispositions–constructs that have been successfully implemented in cross-cultural studies (e.g., Hofer and Chasiotis, 2003; Hofer et al., 2005)–is certainly desirable.

### 1.5 The present research

**Figure 1:**
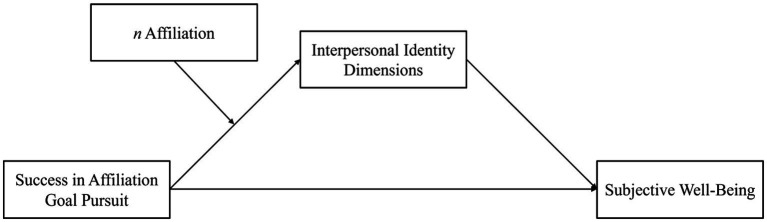
Theoretical model: Success in affiliation goal pursuit is associated with subjective well-being via interpersonal identity dimensions with *n* Affiliation moderating the link between success in affiliation goal pursuit and interpersonal identity dimensions. Interpersonal identity dimensions: commitment, exploration in-depth, and reconsideration of commitment. Subjective well-being: Composite score of life satisfaction, positive and negative affect, and general anxiety.

Given the results on the association between goals, identity commitment, and well-being, we hypothesized that successful realization of affiliation goals predicts higher levels of interpersonal identity commitment and lower levels of identity doubt (i.e., reconsideration of commitment), which both, in turn, positively relate to well-being. Moreover, we assumed to find the positive relationship between successful pursuance of affiliation goals and dimensions of interpersonal identity commitment, particularly among adolescents with a pronounced implicit affiliation motive. We finally hypothesized that the moderating effect of implicit motives is not qualified by adolescents’ culture of origin. Figure 1 depicts the hypothesized moderated mediation model(s) to be tested.

## 2 Materials and methods

### 2.1 Sample

#### 2.1.1 Sample selection

Data for the present analyses were collected within the first measurement point of a longitudinal project on development in adolescence (e.g., Lehmann et al., 2021; Hofer et al., 2022). Samples were gathered from Western individualistic (Germany) and non-Western collectivistic (Zambia) cultural contexts that typically differ in socialization strategies, behavioral norms, beliefs, and value orientations (e.g., Schwartz, 1992; Keller, 2007). Culture is not a static phenomenon; rather, it continuously changes through encounters with other cultures or technical and educational innovations. Nevertheless, Zambians still emphasize values reflecting a strong sense of relatedness and social stability significantly more than Germans; in contrast, German people typically accentuate values reflecting autonomy and openness for change (e.g., Hofer and Chasiotis, 2003; for the present samples, see Lehmann et al., 2021). Thus, this wide range of sociocultural orientations allows testing the equivalence of relationships among variables across cultural contexts (Van de Vijver and Leung, 1997).

Based on ethnic and/or linguistic affiliations, Zambia is a multi-ethnic nation of mainly Bantu-speaking groups. In the 2010 census (Zambian Central Statistical Office, 2012), more than 70 groups were listed. However, most Zambians belong to nine main ethnolinguistic groups (Posner, 2005). In rural areas, each ethnic (linguistic) group is concentrated in a particular geographic region, but in urban areas, particularly in the capital Lusaka, where data collections were conducted, all ethnic groups are present (Kula and Lutz, 2008). As ethnic Bantu groups share significant cultural orientations, recruitment in Zambia was not limited to any specific ethnic/linguistic group. In the German subsample, all participants were born and raised in Germany.

#### 2.1.2 Participants

As reported in literature, moderation effects of implicit motives are generally small (*f*^2^ = 0.02; e.g., Hofer et al., 2010; Hofer and Busch, 2011). Thus, sample size assuring an adequate power to examine moderation effects in the mediational model was inspected: Assuming a small effect size with alpha set to 0.05 (two-tailed testing) and power to 0.80, analyses recommended assessing data from at least 395 participants in each of the cultural samples.

In total, 1,020 adolescents took part in data assessments at the first measurement point. However, 81 data sets (7.9% of the total sample; female participants: *n* = 33) could not be considered in analyses due to an incomplete assessment of implicit motive strengths (missing stories to single picture cues; non-codable stories due to insufficient length of stories) or missing data on goal and identity dimensions, and indicators of well-being. Adolescents who were excluded from analyses did not differ in distributions of sex and class level from adolescents in the final sample. However, a significantly higher percentage of Zambian adolescents (12.1%; *n* = 55) compared to German adolescents (4.6%; *n* = 26) had to be excluded, *χ^2^*_1, 1,020_ = 19.67, *p* < 0.001. Moreover, participants excluded from analyses were slightly older (*M* = 15.27; SD = 1.69) than adolescents included in the final sample (*M* = 14.74; SD = 1.65), *t*_1015_ = 2.70, *p* = 0.007, *d* = 0.32.

Thus, data of 541 German (256 female individuals; 47.3% of the German sample) and 398 Zambian (182 female individuals; 45.7% of the Zambian sample) adolescents that were recruited in grades 7 to 11 at three secondary schools (Germany: Gymnasium) in the southwestern part of Germany and 2 state-funded secondary schools in Lusaka, Zambia, were included in analyses. Cultural samples did not differ in distribution of sex. Adolescents were aged between 11 and 21 years (*M* = 14.74; SD = 1.65). Female and male adolescents and German and Zambian participants, respectively, did not differ in age.

### 2.2 Procedure

We conducted our cross-cultural study in line with the Declaration of Helsinki. Moreover, the Research Ethics Committee of the University of Zambia and the local school authority in Rhineland Palatinate (Germany) approved our study. Before recruiting adolescents, school administrators and parents were informed about the study, parents’ consent was obtained for younger students. Students voluntarily participated, and we assured them that any information given would be treated confidentially and processed anonymously. All participants signed their informed consent forms prior to data assessments. All participants were compensated.

Trained local research assistants supervised sessions. Students took the questionnaire during free periods on school premises. In Germany, measures were administered in German. In Zambia, measures were administered in English, which is one of the official languages, mostly used in administration and higher education. Zambian students were conversant with English and had no difficulty completing the questionnaire. However, local study assistants were present during the data collection to help in case of any problems of comprehension. English and German versions of all measurements were available.

### 2.3 Measures

In addition to data not relevant for present analyses (e.g., parenting styles; self-regulatory capacities), adolescents provided data on the implicit affiliation motive, facets of well-being, interpersonal identity dimensions, and success in pursuance of affiliation goals at the first measurement point.

#### 2.3.1 Implicit affiliation motive (*n* Affiliation)

A picture story exercise (PSE; McClelland et al., 1989), the well-established method for assessing implicit motives, was administered to assess the strength of *n* Affiliation. Following a standard instruction (Smith et al., 1992), the adolescents were informed that they would be viewing several pictures one after another. They were asked to imagine what is going on in the depicted situation and to write a story about the people shown in the picture. It was emphasized that there are no right or wrong stories. Each picture card was shown for 30 s, and the adolescents were given 5 min to write a story on it. On each story sheet, four questions reminded participants to write a complete story: 1. What is happening? Who are the people? 2. What has led up to this situation? How did the story begin? 3. What are the people thinking about, what do they want, and how do they feel? 4. What will happen? How will the story end? (see Smith et al., 1992, for further details on instructions for PSE measurements).

We used six picture cards that have successfully been used in cross-cultural research (e.g., Hofer et al., 2010) in the following order: *couple by a river, ship captain, women in a lab, night club scene, boxer,* and *four men seated at a table* (for reprints, see McClelland and Steele, 1972; McClelland, 1975; Smith, 1992). The picture set has an adequate pull for the target motive to provide a valid and meaningful measure of *n* Affiliation (see Schultheiss and Brunstein, 2001).

Stories were coded for *n* Affiliation following the guidelines set forth in the manual for scoring motive imagery in running text developed by Winter (1994; see also for details on scoring rules). This manual has proven to adequately assess implicit motives in various cultural groups (e.g., Hofer et al., 2010; Hofer and Busch, 2011). *n* Affiliation is defined as a concern for a warm, close relationship with others. According to the manual, the following responses indicating friendly relationships are scored for *n* Affiliation: positive feelings toward others, regret about the disruption of a relationship, friendly companionate activities, or friendly nurturant acts (Winter, 1994).

Stories were scored by four well-experienced German assistants who all achieved percentage agreements of 85% or higher with training materials prescored by experts (Winter, 1994). All assistants initially coded 20 full sets of picture stories: To determine the level of interrater reliability, intraclass correlations (ICC; Shrout and Fleiss, 1979) were calculated by applying a two-way, random-effects model with absolute agreement. ICC for *n* Affiliation was 0.85, indicating a good interrater reliability (single measure reliability). Consequently, each assistant independently coded a different set of the remaining picture stories. Scoring problems were discussed and resolved in regular team meetings.

Across stories, the total number of affiliation motive imageries ranged from 0 to 16 (*M* = 4.47; SD = 2.42). Word count which ranged from 184 to 786 words (*M* = 406.69; SD = 104.26) was significantly correlated with a number of motive images, *r* = 0.41, *p* < 0.001. Thus, the effects of word count on motive scores were corrected by regression across cultural groups.

#### 2.3.2 Facets of (subjective) well-being

In total, three well-established methods were administered to assess data on components of individuals’ well-being: (A) the Satisfaction With Life Scale (SWLS; Diener et al., 1985), (B) the hedonic scale of the UWIST Mood Adjective Checklist (UMACL; Matthews et al., 1990), and (C) the General Anxiety Disorder Subscale (GAD) taken from the Screen for Child Anxiety-Related Emotional Disorders (SCARED; Birmaher et al., 1997).

- The SWLS is a measure of global satisfaction with life widely used in cross-cultural research. In total, 5 Items (e.g., *In most ways, my life is close to my ideal*) are rated on a 7-point Likert scale (1 = strongly disagree to 7 = strongly agree). The level of life satisfaction is indicated by the mean of the five ratings. Cronbach’s alphas for measurements of life satisfaction were 0.73 (total sample), 0.82 (German sample), and 0.61 (Zambian sample).
- The hedonic scale of the UMACL consists of 12 items measuring two primary mood dimensions, that is, positive (6 items; e.g., *pleased*) and negative affect (6 items; e.g., *depressed*) of subjective well-being. In our study, we asked participants to indicate on a 5-point Likert scale ranging from 1 (never) to 5 (always) to what extent they experience each of the mood states in general. Due to missing data (more than one item used for scale development is missing), a negative affect score could not be calculated for one German participant. Cronbach’s alphas were 0.72 (total sample), 0.81 (German sample), and 0.57 (Zambian sample) for positive affect and 0.87 (total sample), 0.89 (German sample), and 0.55 (Zambian sample) for negative affect.
- The GAD subscale represents a measure of individuals’ level of general anxiety. The subscale includes nine items (e.g., *I worry about other people liking me*). Adolescents indicated on a 5-point Likert scale (1 = not true; 5 = very true) how well the statements describe them. Cronbach’s alphas were 0.80 (total sample), 0.86 (German sample), and 0.63 (Zambian sample).

#### 2.3.3 Interpersonal identity dimensions

The Utrecht-Management of Identity Commitments Scale (U-MICS; Crocetti et al., 2008) was administered to assess data on dimensions of interpersonal identity formation. The U-MICS includes three identity dimensions: commitment (five items), in-depth exploration (five items), and reconsideration of commitment (three items). Each item is evaluated on a 5-point Likert scale, ranging from 1 (completely untrue) to 5 (completely true). The items of the U-MICS can be adapted to assess identity dimensions in different life domains. In the present study, we collected data on interpersonal identity formation in the subdomains of *best friend*, *peers*, and *romantic partner*. Given that only those adolescents in a steady relationship responded to items of the subdomain *romantic partner*, it is not used in current analyses. Moreover, analyses focus on the subdomain *best friend* because it represents a key component in which interpersonal identity work must be done (see, e.g., Crocetti et al., 2008; see footnote 7 for findings related to the subdomain *peers*). Cronbach’s alphas were 0.82 (total sample), 0.84 (German sample), and 0.82 (Zambian sample) for the dimension commitment (e.g., *My best friend gives me self-confidence*); 0.66 (total sample), 0.59 (German sample), and 0.73 (Zambian sample) for the dimension exploration in-depth (e.g., *I often try to find out what other people think about my best* friend); and 0.81 (total sample), 0.79 (German sample), and 0.82 (Zambian sample) for reconsideration of commitment (e.g.*, I often think it would be better to try to find a different best friend*).^2^

#### 2.3.4 Affiliation goals

Successful realization of affiliation goals was assessed by the questionnaire GOALS (Pöhlmann and Brunstein, 1997). Among other goal items (e.g., power, achievement, and altruism), adolescents rated four affiliation goals on a 5-point Likert scale (1 to 5) with higher scores indicating more success in realization. The four affiliation items are as follows: *engage in a lot of activities with friends*, *spend a lot of time with friends*, *have a wide circle of friends*, and *be friends with many people*. In the total sample, Cronbach alphas were 0.84 for successful affiliation goal realization (German sample: 0.86; Zambian sample: 0.77).^3^,^4^

### 2.4 Measurement invariance

Cross-cultural research requires testing of measurement equivalence. Referring to the measurement of *n* Affiliation, construct equivalence and meaningfulness have been proven in diverse cultural contexts (see, e.g., Hofer et al., 2005). Most picture stimuli used for the assessment of *n* Affiliation in the study at hand have been successfully applied in past cross-cultural research on implicit motives (e.g., Hofer et al., 2010). However, evidence on the applicability of PSE methods in cross-cultural research is still scarce. Thus, in line with recommendations provided by Van de Vijver and Leung (1997), we scrutinized our picture set for item/picture bias (i.e., differential item functioning) using analysis of variance (IBM SPSS Statistics, version 27).

In analyses, the single item/picture score for *n* Affiliation was the dependent variable, and cultural context (two levels) and score level (three levels) were used as factors. The total score for *n* Affiliation across the six picture cues was divided into three evenly sized score-level groups (low–medium–high) to determine the score level. In analyses, a significant effect of the score level is assumed to be found as individuals at higher score levels ought to score higher on a given picture cue. In contrast, either pronounced effects of cultural context (uniform bias) or its interaction with the score level (non-uniform bias) would indicate bias (Van de Vijver and Leung, 1997).

As assumed, the score level (i.e., motive strength) significantly predicts the score of *n* Affiliation for each of the six picture cards (*F*s_2, 933_ range from 19.73 to 194.51; *p*s < 0.001; *η*^2^s range from 0.041 to 0.294). While uniform bias was absent in analyses on four picture cards (*F*s_1, 933_ ≤ 2.02; *p*s ≥ 0.156; *η*^2^s ≤ 0.002), cultural group significantly predicted the level of *n* Affiliation scored for *night club scene* (*F*_1, 933_ = 4.08; *p* = 0.044; *η*^2^ = 0.004) and *four men seated at a table* (*F*_1, 933_ = 13.07; *p* < 0.001; *η*^2^ = 0.014). Finally, the findings indicated the absence of non-uniform bias in analyses on four picture cues effect (*F*s_2, 933_ ≤ 1.27; *p*s ≥ 0.281; *η*^2^s ≤ 0.003), but the interaction term was significant in analyses on *couple by a river* (*F*_2, 933_ = 5.69; *p* = 0.004; *η*^2^ = 0.012) and *four men seated at a table* (*F*_1, 933_ = 4.12; *p* = 0.016; *η*^2^ = 0.009). However, none of the effects apparently pointing to uniform or non-uniform bias, respectively, is large enough to be practically important (η^2^ ≥ 0.06; Meiring et al., 2005). Thus, we could refrain from removing any of the picture cues in our set.

Using AMOS (version 29), we applied multigroup confirmatory factor analysis (CFA) to examine measurement equivalence of self-reports on facets of subjective well-being (life satisfaction and positive/negative affect), general anxiety, affiliation goals (success), and identity dimensions (interpersonal domain of best friend). In this process, we conducted three separate analyses using data on (a) identity dimensions, (b) components of subjective well-being, and (c) general anxiety and affiliation goals (success). In multigroup CFAs, at least two psychological constructs were considered conjointly as it is not advisable to scrutinize measurement equivalence of single scales (e.g., Anderson and Gerbing, 1988; see for example, information on discriminant validity and covariance of constructs). In each of the analyses, configural invariance (equivalence of measured constructs) and measurement weights invariance (equivalence of factor loadings) were examined. The latter is considered a prerequisite to test the equivalence of relationships among psychological constructs across cultural groups but not (cultural) group differences in mean levels of psychological constructs (see Van de Vijver and Leung, 1997, for details on structure- and level-oriented approaches in research). Internal consistencies of scales indicate that item sets can be considered as measuring single latent variables across cultural samples at hand; thus, none of the items ought to be excluded *a priori* from CFA. In each of our analyses, the ratio of cases/observations to a number of parameters to be estimated was above 10, a threshold typically recommended in the literature (e.g., Kline, 1998).

In the multigroup CFAs, we tested and compared two increasingly restrictive measurement models to each other, that is, an unconstrained model with no equality constraints across cultural groups (i.e., screening equivalence of factor numbers and item patterns) and a measurement weights model with measurement weights constrained to be equal across cultural groups (i.e., screening equivalence of item content and psychometric properties; variances and covariance of the latent scores estimated separately for each group). The fit of two nested models was evaluated from the perspective of multiple fit indices. Hence, a measurement invariance hypothesis should not be rejected when a change in fit indices from the unconstrained to the constrained model is low (e.g., *∆*GFI ≤ −0.01; *∆*RMSEA ≤0.015; see Cheung and Rensvold, 2002; Chen, 2007).

First, measurement invariance of identity dimensions was tested (unconstrained model: 182 data points and 58 unknown parameters to be estimated). The unconstrained model adequately fits the data (e.g., *χ*^2^ = 493.96; *df* = 124; GFI = 0.918; RMSEA = 0.058). In both cultural samples, all items were significantly loaded on the specified factor (factor loadings ranging from 0.31 to 0.79; critical ratios >5.28; *p*s < 0.001). Moreover, the implementation of constraints on factor loadings indicates a negligible increment of model fit (∆GFI = −0.011; ∆RMSEA = 0.001).

Then, the measurement invariance of components of subjective well-being, that is, life satisfaction and both mood states, was inspected (unconstrained model: 306 data points and 74 unknown parameters to be estimated). Again, the unconstrained model adequately fits the data (e.g., *χ*^2^ = 457.55; *df* = 232; GFI = 0.939; RMSEA = 0.034) with items significantly loading on the specified factor in both cultural samples (factor loading 0.17 to 0.80; critical ratios >3.38; *p*s < 0.017). Constraining factor loadings to be equal across cultural samples did not result in a significant increment of model fit (∆GFI = −0.009; ∆RMSEA = 0.003).

Finally, measurement invariance of the model including general anxiety and self-reported success in the realization of affiliation goals (unconstrained model: 156 data points and 50 unknown parameters to be estimated) was tested. The results show that the unconstrained model adequately fits the data (e.g., *χ*^2^ = 263.11; *df* = 106; *GFI* = 0.954; RMSEA = 0.041); all items significantly loaded on the specified factor in both cultural samples (factor loadings 0.27 to 0.85; critical ratios >3.56; *p*s < 0.001). Constraining factor loading to be equal across cultural samples did not significantly impair the model fit (∆GFI = 0.002; ∆RMSEA = −0.001).

To conclude, the results clearly point to configural and metric measurement invariance: Psychological constructs evidently overlap between cultural samples. Thus, (structural) relationships among constructs can meaningfully be analyzed across cultural groups.

### 2.5 Developing an index of individuals’ well-being

To develop an index of well-being, correlations among measures of subjective well-being and general anxiety were inspected. In the total sample, correlations were highly significant (*p*s < 0.001). In detail, life satisfaction significantly related to positive affect (*r* = 0.47), negative affect (*r* = −0.45), and general anxiety (*r* = −0.37). Self-reported positive affect was negatively associated with both negative affect (*r* = −0.38) and general anxiety (*r* = −0.23). The latter were positively related to each other (*r* = 0.46).^5^ Moreover, principal components analysis based on the four measures of well-being produced a single factor that accounted for 54.6% of the variance. Factor loadings were 0.79 (SWLS), 0.70 (positive affect), −0.79 (negative affect), and − 0.68 (general anxiety). Thus, the factor score was used in analyses as a single indicator of well-being.

## 3 Results

The following results are subdivided into three main sections: First, descriptive statistics and correlations among variables, as well as their relation to age and sex are provided. Then, analyses of the assumed moderated mediation model across cultural samples are presented. Finally, the findings are provided on the effect of culture on the assumed moderation effect of *n* Affiliation on the link between goal success and identity.

### 3.1 Descriptive statistics, correlations, and covariates

In Table 1, descriptive statistics of measures for both cultural samples are presented. Correlations among constructs and with age and sex for the total sample are given in Table 2.

**Table 1:** Descriptive statistics.

	*M* (SD) (GER)	*M* (SD) (ZAM)
1 *n* Affiliation	0.30 (2.43)	−0.40 (1.78)
2 Affiliation goals^1^	3.56 (0.87)	3.01 (0.91)
3 Commitment^2^	3. 50 (0.76)	3.65 (0.79)
4 In-depth exploration^2^	3.10 (0.61)	3.48 (0.76)
5 Reconsideration of commitment^2^	1.61 (0.73)	2.12 (0.97)
6 Well-being index	0.33 (1.05)	−0.45 (0.73)

^1^Success in goal realization; ^2^Identity dimensions.

**Table 2:** Correlations among measurements and with participants’ age and sex.

	1	2	3	4	5	6
1 *n* Affiliation	---					
2 Affiliation goals^1^	0.17	---				
3 Commitment^2^	0.05	0.19	---			
4 In-depth exploration^2^	0.02	0.09	0.55	---		
5 Reconsideration of commitment^2^	−0.06	−0.12	−0.27	0.06	---	
6 Well-being index	0.15	0.39	0.10	−0.13	−0.28	---
7 Age	−0.05	−0.15	0.05	0.05	0.01	−0.18
8 Sex^3^	−0.14	0.11	−0.18	−0.18	0.14	0.09

≥|0.09| *p* < 0.01; ≥|0.12| *p* < 0.001. ^1^Success in goal realization; ^2^Identity dimensions; ^3^Sex coded ^0^female, ^1^male.

As indicated, *n* Affiliation shows weak but significant relationships with both successful realization of affiliation goals and well-being. Successful goal realization significantly relates to higher scores of commitment, in-depth exploration, and well-being but lower scores for reconsiderations of commitment. Commitment significantly relates to high levels of well-being and in-depth exploration but lower levels of reconsideration of commitment. Finally, well-being shows significant negative associations with the identity dimensions of in-depth exploration and reconsideration of commitment.^6^

Analyses also indicate that a higher age relates significantly to lower levels of success in goal realization and well-being. Moreover, participants’ sex significantly relates to all constructs under consideration: Compared to male adolescents, female adolescents report higher levels of *n* Affiliation, identity commitment, and in-depth exploration but lower levels of successful goal realization, reconsideration of commitment, and well-being.

The paths linking goal realization, identity dimensions, and well-being are integral parts of the model to be tested (see Figure 1). To observe whether the model is cross-culturally equivalent, these paths will be tested for differences in magnitude between cultural samples in the final model analyses. Thus, we could refrain from testing whether corresponding pairs of correlations significantly differed from one another at this stage. However, we tested for cultural differences in correlations including *n* Affiliation and associations between identity dimensions. Analyses verified that *n* Affiliation and successful realization of affiliation goals are more closely related to each other in the German (*r* = 0.19; *p* < 0.001) than in the Zambian subsample (*r* = 0.05; n. s.; *z* = 2.04; *p* = 0.041). Similarly, *n* Affiliation and the well-being index show a significant positive association among German (*r* = 0.19; *p* < 0.001) but not among Zambian adolescents (*r* = −0.06; n. s.; *z* = 3.11; *p* = 0.002). The size of *n* Affiliation’s correlations with the identity dimensions does not differ between samples (*z*s ≤ 1.38; *p*s ≥ 0.180). With respect to identity dimensions, the associations between reconsideration of commitment and both commitment and in-depth exploration do not differ in strength in both subsamples (*z*s ≤ 0.87; *p*s ≥ 0.384). However, in-depth exploration and commitment are more closely related in the Zambian (*r* = 0.61; *p* < 0.001) than in the German sample (*r* = 0.49; *p* < 0.001; *z* = −2.69; *p* = 0.007).

### 3.2 Test of models on moderated mediation across cultural groups

The assumed models were tested by employing the PROCESS macro for SPSS (version 4.0; see Hayes, 2022). In the first step of analyses, the template for moderated mediation (model 7) was used with successful realization of affiliation goals (predictor), identity dimensions (mediators), well-being index (dependent variable), and *n* Affiliation (moderator). Age, sex, and culture were entered as covariates. Variables entered in interaction terms were mean-centered.

In model A, we tested the assumption that successful realization of affiliation goal predicts, particularly among adolescents high in *n* Affiliation, higher levels of identity commitment which, in turn, relates to a higher degree of well-being. In model B, successful pursuance of affiliation goals, above all among adolescents with a strong *n* Affiliation, is assumed to relate to lower levels of identity doubt, that is, reconsideration of commitment, which, in turn, relates negatively to well-being. Finally, in the exploratory model C, as no specific hypotheses were developed, we tested the effects of in-depth exploration on the link between goal success and well-being. The findings of first-step analyses are given in Table 3.

**Table 3:** Moderated mediation of successful pursuance of affiliation goals on well-being.

	Model A		Model B		Model C	
Path A (on identity dimension^1^) *F*-value (df); *R*^2^	21.05*** (6, 931)	0.12	18.99*** (6, 931)	0.11	27.98*** (6, 931)	0.15
Coefficient estimates	b	S.E.	b	S.E.	b	S.E.
Affiliation goals	0.24***	0.03	−0.07*	0.03	0.17***	0.03
*n* Affiliation	0.00	0.01	0.01	0.01	0.00	0.01
Affiliation goals * *n* Affiliation	0.03*	0.01	−0.04*	0.01	0.00	0.01
Age	0.05**	0.02	−0.01	0.02	0.04**	0.01
Sex	−0.33***	0.05	0.24***	0.06	−0.30***	0.04
Culture	−0.30***	0.05	−0.46***	0.06	−0.48***	0.05
Path B (on well-being index) F-value (df); *R*^2^	67.51*** (5, 932)	0.27	74.47*** (5, 932)	0.29	64. 90*** (5, 932)	0.26
Affiliation goals	0.26***	0.03	0.28***	0.03	0.31***	0.03
Identity dimension^1^	0.14**	0.04	−0.21***	0.03	−0.08^†^	0.04
Age	−0.09***	0.02	−0.09***	0.02	−0.08***	0.02
Sex	0.19**	0.06	0.20***	0.06	0.12*	0.06
Culture	0.66***	0.06	0.52***	0.06	0.58***	0.06

^†^*p* < 0.10; *p < 0.05; **p < 0.01; ***p < 0.001. ^1^Commitment (Model A), reconsideration of commitment (Model B), and in-depth exploration (Model C) represent mediators in analyses.

**Figure 2:**
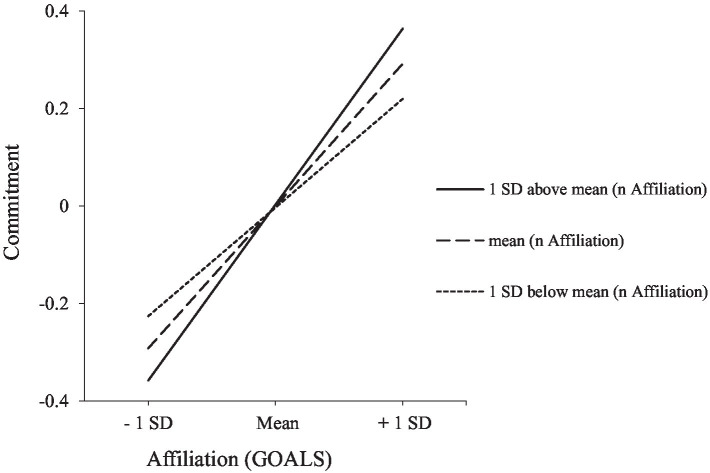
Effects of *n* Affiliation on the link between affiliation goals and commitment.

Model A: Referring to path A, coefficient estimates shown in Table 3 indicate that there is a significant link between success in affiliation goals and identity commitment in addition to the effects of participants’ characteristics (i.e., identity commitment is associated with an older age, female sex, and being Zambian). This main effect, however, is qualified by the strength of *n* Affiliation (*F*_change, 1, 931_ = 4.94, *R^2^*_change_ = 0.01, *p* = 0.027). An inspection of conditional effects at three values of the moderator (i.e., the mean and at one standard deviation above and below the mean of *n* Affiliation) shows that there are significant associations between goal success and identity commitment at all three values of the moderator; however, the associations become stronger at higher levels of *n* Affiliation: *B* = 0.19; *SE* = 0.04; *t* = 5.20; *p* < 0.001 for low *n* Affiliation; *B* = 0.24; *SE* = 0.03; *t* = 8.62; *p* < 0.001 for medium *n* Affiliation; *B* = 0.30; *SE* = 0.04; *t* = 7.52; *p* < 0.001 for high *n* Affiliation (see Figure 2).

Concerning path B, analyses point again to the effects of age (younger age), sex (male sex), and culture (German origin) on well-being. In addition, a significant direct effect of goal success on well-being was verified. Finally, higher levels of commitment predicted enhanced levels of well-being. Conditional indirect effects show that there are significant indirect effects of goal success via identity commitment on well-being at all levels of *n* Affiliation, yet indirect effects become stronger at higher levels of the moderator (*n* Affiliation low: indirect effect = 0.026; S.E. = 0.010; 95% confidence interval (CI) based on 5,000 bootstrap resamples = 0.009 to 0.046; medium: indirect effect = 0.034; S.E. = 0.011; CI = 0.013 to 0.056; high: indirect effect = 0.042; S.E. = 0.014; CI = 0.015 to 0.072). Finally, the index of the moderated mediation is small but significant (index = 0.004; SE = 0.002; CI = 0.0002 to 0.009).

**Figure 3:**
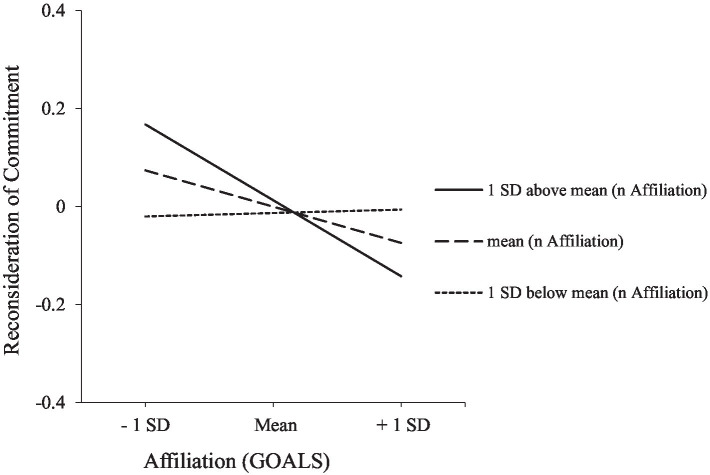
Effects of *n* Affiliation on the link between affiliation goals and reconsideration of commitment.

Model B: With respect to path A, coefficient estimates indicate that higher levels of goal success are related to lower levels of reconsideration of commitment, in addition to sex- and culture-related effects (i.e., identity doubt is more pronounced among male and Zambian adolescents, respectively). Again, this association is moderated by the strength of *n* Affiliation (*F*_change, 1, 931_ = 6.62, *R^2^*_change_ = 0.01, *p* = 0.010; see Figure 3): While goal success does not relate to level of identity doubt among adolescents low in *n* Affiliation (*B* = 0.01; SE = 0.04; *t* = 0.17; *p* = 0.869), significantly negative associations are shown at medium (*B* = −0.06; SE = 0.03; *t* = −2.02; *p* = 0.044) and high levels of *n* Affiliation (*B* = −0.14; SE = 0.05; *t* = −3.12; *p* = 0.002).

Concerning path B, analyses indicate the effects of age (younger age), sex (male sex), and culture (German origin) on well-being. Moreover, a significant direct effect of goal success on well-being was verified. Finally, lower levels of identity doubt predicted higher levels of well-being. However, conditional indirect effects only point to a significant indirect effect of goal success via identity doubt on well-being at high levels of *n* Affiliation (indirect effect = 0.030; S.E. = 0.011; CI = 0.009 to 0.053) but not at medium (indirect effect = 0.013; S.E. = 0.008; CI = −0.002 to 0.029) and low levels of the moderator (indirect effect = −0.001; S.E. = 0.009; CI = −0.018 to 0.016). Again, the index of the moderated mediation is small but meaningful (index = 0.007; SE = 0.003; CI = 0.0002 to 0.013).

Model C: Coefficient estimates related to path A indicate that higher levels of goal success are related to higher levels of in-depth exploration, in addition to the age-, sex- and culture-related effects (i.e., in-depth exploration is more pronounced among older, female, and Zambian adolescents, respectively). Analyses do not point to a moderation effect of *n* Affiliation (*F*_change, 1, 931_ = 0.16, *R^2^*_change_ < 0.001, *p* = 0.693).

Over and above the effects of age (younger age), sex (male sex), and culture (German origin), analyses on path B indicate a significant direct effect of goal success on well-being and a marginally significant positive effect of in-depth exploration. However, analyses do not point to indirect effects at any level of the moderator (low: indirect effect = −0.013; S.E. = 0.008; CI = −0.032 to 0.001; medium: indirect effect = −0.014; S.E. = 0.009; CI = −0.033 to 0.001; high: indirect effect = −0.014; S.E. = 0.009; CI = −0.036 to 0.001). Similarly, the moderated mediation index is insignificant (index = −0.0003; SE = 0.001; CI = −0.003 to 0.002).

### 3.3 Effects of culture on model links

In the final step of analyses (models D to F), we tested whether culture qualifies the moderation effect of *n* Affiliation on the link between affiliation goals and commitment-related identity dimensions (i.e., path A in Models A to C) by implementing the PROCESS model 3. As culture is used as an additional moderator, only participants’ age and sex were entered as covariates into the model. The results are presented in Table 4. In the text, we will only refer to the significant effects of culture on assumed associations between psychological constructs.

**Table 4:** Effects of culture on the assumed moderation of *n* Affiliation.

	Model D^1^		Model E^1^		Model F^1^	
F-value (df); *R*^2^	14.42*** (9, 929)	0.12	15.20*** (9, 929)	0.13	19.91*** (9, 929)	0.16
Coefficient estimates	b	S.E.	b	S.E.	b	S.E.
Affiliation goals	0.24***	0.03	−0.08*	0.03	0.17***	0.03
*n* Affiliation	−0.00	0.01	0.01	0.01	0.00	0.01
Affiliation goals * *n* Affiliation	0.02^†^	0.01	−0.03*	0.02	0.01	0.01
Culture	−0.30***	0.05	−0.47***	0.06	−0.50***	0.05
Affiliation goals * culture	0.08	0.06	−0.28***	0.06	−0.07	0.05
*n* Affiliation * culture	−0.01	0.03	0.03	0.03	−0.04	0.02
Affiliation goals * *n* Affiliation * culture	0.04	0.03	−0.02	0.03	0.04	0.02
Age	0.05**	0.02	−0.01	0.02	0.04**	0.01
Sex	−0.33***	0.05	0.24***	0.06	−0.31***	0.04

^†^*p* < 0.10; *p < 0.05; **p < 0.01; ***p < 0.001. ^1^Commitment (Model D), reconsideration of commitment (Model E), and in-depth exploration (Model F) represent mediators in analyses.

In general, the findings given in Table 4 are in line with results obtained in analyses across cultural groups (see Table 3; path A). Above all, moderation effects of *n* Affiliation do not seem to be qualified by culture (Model D: *F*_1, 929_ = 1.85; *p* = 0.175; *R^2^*_change_ = 0.002; Model E: *F*_1, 929_ = 0.56; *p* = 0.455; *R^2^*_change_ < 0.001; Model F: *F*_1, 929_ = 2.59; *p* = 0.108; *R^2^*_change_ = 0.002): The interaction effect is still insignificant in analyses on in-depth exploration (*p* = 0.524) and remains significant in analyses on identity doubt (*p* = 0.028). In analyses on commitment, the interaction is still marginally significant (*p* = 0.072). Although the level of significance does not reach *p* < 0.05, interaction effects are typically considered to be meaningful at *p* < 0.10 as detecting interaction effects is typically associated with several problems (e.g., an increased chance of committing a type II error; Pedhazur, 1997; Fairchild and MacKinnon, 2009). Only one additional moderating effect of culture was identified: There was a negative association between successful pursuance of affiliation goals and reconsideration of commitment in the German sample (*r* = −0.20; *p* < 0.001), but a positive association among Zambian adolescents (*r* = 0.11; *p* = 0.026).^7^

## 4 Discussion

Focusing on the interpersonal (affiliative) domain, we aimed to examine the relationship between personal goals, identity development, implicit motives, and subjective well-being in two divergent cultural contexts. We hypothesized that commitment-related facets of identity development mediate the link between successful realization of goals and well-being, particularly among adolescents characterized by a strong implicit affiliation motive.

In initial analyses, the cross-cultural applicability of the measurements at hand could be verified. Based on these bias-free measures, we found conclusive evidence for the validity of our assumed moderated mediation model when considering commitment dimensions of interpersonal identity. As reported in extant research, successful realization of affiliation goals predicts well-being (e.g., Klug and Maier, 2015). Moreover, both interpersonal identity commitment and reconsideration of commitment mediate the link between goal success and well-being: Affiliation goal success is associated with higher scores of commitment and lower scores of reconsideration of commitment, which both meaningfully predict levels of well-being. Our findings on the association between identity dimensions are in line with the results reported by Crocetti et al. (2008) who found that higher levels of anxiety and depressive symptoms relate to low levels of commitment but higher levels of both in-depth exploration and reconsideration of commitment.

Most importantly, the results confirm the assumed moderation effect of *n* Affiliation: Indirect effects of goal success on well-being via identity commitment and reconsideration of commitment in the interpersonal domain, respectively, become stronger (commitment) the higher *n* Affiliation and are only present (reconsideration of commitment) when *n* Affiliation is well pronounced. Among adolescents low in *n* Affiliation goal success does not predict reconsideration of interpersonal identity commitments at all. Thus, when realizing so-called hot goals (Schultheiss et al., 2008), individuals proceed more easily in their identity development. They may establish stronger interpersonal identity commitments and seem to be more content with their commitments (Hofer et al., 2006).

The realization of motive-congruent goals enables individuals to experience enhanced levels of well-being, presumably triggered by the consummation of positive affective experiences when satisfying implicit motive dispositions (Schultheiss et al., 2008). Such experiences may also be intrinsically rewarding and help individuals to get a taste of their true selves. Consequently, they are more likely to commit to personally expressive identity elements (Waterman, 2011) that also include motive-congruent life goals. In contrast, commitment to goals that reflect others’ demands and are likely to be at odds with one’s implicit motives relates to emotional distress (Brunstein et al., 1998). Thus, commitment to such (identity) goals is rather unlikely. Moreover, existing commitments might be reconsidered, and individuals start (again) to look for other identity options. Thus, implicit motives might play a significant role in weighing disposition when individuals search for meaningful elements of their identity.

Finally, exploratory analyses with respect to in-depth exploration neither produced evidence for a moderating effect of *n* Affiliation on the link between affiliation goal success and interpersonal in-depth exploration nor for a mediating effect of this identity dimension. It was found that the more the adolescents successfully realized their affiliation goals, the higher the levels of in-depth exploration they reported. Thus, successful goal pursuance generally may foster individuals’ willingness to explore identity options. However, such (continuous) exploration, which is indispensable to learning more about identity elements linked to existing commitments, seems to be a double-edged sword (Crocetti et al., 2008): While in-depth exploration can strengthen the commitment to identity elements, it also can be a dead end if no decisions on commitments can be made, and identity elements are continuously explored in a rather ruminative way. This double nature of in-depth exploration might also explain its negative association with well-being and the lack of correlation with reconsideration of commitment.

Additional analyses on the role of culture clearly proved that relationships between variables and moderating effects of *n* Affiliation are not qualified by adolescents’ cultural background. These findings represent another example of universal effects of implicit motives on individuals’ psychological and developmental processes (see Hofer, 2010, for an overview). Only the relationship between affiliation goal success and reconsideration of commitment is qualified by culture. As expected, higher levels of goal success are associated with lower levels of reconsideration of commitment among German adolescents, indicating individuals’ satisfaction with existing goal commitments. In contrast, a weak but significantly positive association between goal success and reconsideration of commitment is found in the Zambian sample. Although speculative, it might be that Zambian adolescents, who grow up in a collectivistic environment, continue to explore interpersonal identity options based on a sense of security provided by already established social relationships. However, future research is needed to foster this line of argument.

In addition to associations between psychological constructs tested in the assumed moderated mediation models, significant positive relationships between *n* Affiliation and both, goal success and well-being, are present. Therefore, the link between *n* Affiliation and goal success is more pronounced in the German sample, and the association between *n* Affiliation and well-being is significant only in the German sample. While the culture-bound association between *n* Affiliation and well-being deserves closer attention in future research to be clarified, the link between implicit motive strength and goal progress suggests a facilitating effect of implicit motivation–even when a strong sense of goal commitment is missing, individuals can reap effective rewards associated with need fulfillment (Schultheiss et al., 2008). Finally, intercorrelations of identity dimensions corroborate findings reported by Crocetti et al. (2008): Identity commitment positively relates to in-depth exploration and negatively relates to reconsideration of commitment. Moreover, similar associations between identity dimensions were found in both cultural samples. Only the link between commitment and in-depth exploration is more pronounced in the Zambian subsample, although significant in both subsamples.

Additionally, with respect to interpersonal identity dimensions, a positive age effect is found: The older adolescents are the higher levels of identity commitment and in-depth exploration they report. This age-related change illustrates ongoing progress in identity formation from early to late adolescence. Adolescents’ age is also negatively related to their success in goal realization and well-being. Although the present study did not focus on this, analyses indicate sex-related differences in all psychological measurements: Female adolescents report higher levels of *n* Affiliation, identity commitment, and in-depth exploration but lower levels of successful goal realization, reconsideration of commitment, and well-being. While the higher level of *n* Affiliation of female adolescents is in line with the findings reported in literature (Drescher and Schultheiss, 2016), lower levels of self-reported success in the realization of affiliation goals indicate that, even if putting more emphasis on interpersonal relationships, female adolescents perceive it as more difficult to satisfy these needs. Although findings on gender differences in identity development in adolescence are mixed, sex-related differences in identity dimensions mirror an often-reported developmental advantage of women in adolescence (Schwartz et al., 2011).

Finally, analyses point to differences among German and Zambian adolescents in well-being and levels of identity dimensions. Although some cultural differences in identity dimensions might reflect developmental affordances in individualistic Western and collectivistic non-Western cultural contexts (e.g., a more pronounced commitment in collectivistic cultural contexts), we refrain from discussing these mean differences in detail as our analyses on measurement equivalence do not allow such comparisons.

### 4.1 Limitations and outlook

Although our analyses revealed findings in line with our hypotheses, the potential limitations of our study must be addressed. First, the generalizability of our findings is limited as participants were recruited in only two cultural contexts. Moreover, all participants were attending secondary schools in urban areas at the time when data were collected. Thus, our samples neither cover the range of cultural contexts nor are they representative of the German or Zambian cultural context. Thus, future research ought to strive to recruit more representative adolescent samples by including participants from various socioeconomic, educational, and cultural backgrounds. Preferably, our moderated mediation model ought to be examined longitudinally to examine the quality of our proposed (temporal) order of constructs. Currently, one could argue for alternative sequences of processes, for example, the happier the people are, the more successful they might evaluate their goal progress, which might affect their identity development. Although it seems to be more difficult to match motives with ideological identity domains, future research ought to examine the interplay of goals, motives, identity dimensions, and well-being in other identity domains in which adolescents have to make decisions on identity options. Finally, it must be acknowledged that Cronbach alphas are rather moderate for some of the measures in Germany (exploration in-depth) and Zambia (facets of well-being), even if one accepts a threshold of α = 0.6 through 0.7, indicating an acceptable level of reliability. Although unreliable data do not always negatively affect the study results (e.g., attenuation of score correlation) (Nimon et al., 2012), one might argue that the items do not adequately capture the construct in respective subsamples, particularly affect scales in Zambia and the exploration in-depth identity dimension. However, it is important to keep in mind, also with respect to measurements of facets of well-being among Zambian adolescents, that multigroup CFAs clearly supported the assumed factor structure of the respective scales. In addition to indices of reliability, the meaningfulness of constructs ought to be evaluated by indicators of discriminant and convergent validity. Our analyses show that measures of exploration in-depth and facets of well-being meaningfully correlate with other constructs in both cultural samples. Thus, despite rather low alphas for some scales, we are confident that our analyses depict valid associations between psychological constructs in both cultural samples. Given the moderate Cronbach’s alphas of measures of well-being among Zambian adolescents, different instruments that grasp Western and non-Western facets of components of subjective well-being ought to be used in future cross-cultural studies.

### 4.2 Conclusion

Despite these limitations, the present study conducted in two diverse cultural contexts adds to our understanding of the relationship between personal goals, identity development, and well-being and the effects of implicit motives on these relationships. The present study thus contributes to the steadily increasing evidence that implicit motives seem to have comparable (moderating) effects, irrespective of cultural contexts. Hopefully, our study will encourage further cross-cultural research on determinants and consequences of identity formation to enhance our knowledge on universal and culture-bound factors of development.

## Data availability statement

The datasets presented in this study can be found in online repositories. The names of the repository/repositories and accession number(s) can be found at: Data for the present analyses are available in the Open Science Framework: https://osf.io/we6b7.

## Ethics statement

The Research Ethics Committee of the University of Zambia and the local school authority in Rhineland Palatinate (Germany) approved our study. School administrators and parents were informed about the study; parents of younger students were asked for permission. Students voluntarily participated and were assured that any information given would be treated confidentially and processed anonymously. All participants signed an informed consent form prior to data assessments. The studies were conducted in accordance with the local legislation and institutional requirements. Written informed consent for participation in this study was provided by the participants’ legal guardians/next of kin.

## Author contributions

JH: Conceptualization, Formal analysis, Funding acquisition, Investigation, Project administration, Supervision, Writing – original draft. HB: Funding acquisition, Investigation, Project administration, Supervision, Writing – original draft. AM: Investigation, Project administration, Supervision, Writing – review & editing.

## Footnotes

^1^The study was not pre-registered.

^2^The applicability of the U-MICS (Crocetti et al., 2008) for the assessment of dimensions of interpersonal identity formation in Zambian adolescents was tested in a pilot study with 229 Zambian adolescents recruited in a secondary school in Lusaka (grades 9 and 12; *M* = 16,27; SD = 2.40; 76 female students). CFA findings indicate a good fit of the three-factor model in the Zambian sample. Specifically, the following indices were found for the interpersonal domain *best friend* (unconstrained model with 91 data points and 29 unknown parameters to be estimated): *χ^2^* = 101.66; *df* = 62; *GFI* = 0.932; *RMSEA* = 0.056. All items significantly loaded on the specified factor with loadings ranging from 0.35 to 0.74 (critical ratios >3.98; *p*s < 0.001). Cronbach’s alphas for the domain *best friend* were 0.75 (commitment), 0.59 (exploration in-depth), and 0.71 (reconsideration of commitment). For the *peers* domain, the three-factor model also had a good fit: *χ^2^* = 116.61; *df* = 62; *GFI* = 0.921; *RMSEA* = 0.066. Cronbach’s alphas were 0.79, 0.68, and 0.78 for commitment, exploration in-depth, and reconsideration of commitment, respectively. Given the good fit of the three-factor model and the acceptable internal consistency of most scales (in-depth exploration for the domain *best friend* just misses the threshold of 0.6 but internal consistency could not be increased by exclusion of items; see, however, Crocetti et al. (2015), who report that “exploration in-depth” often shows lower Cronbach’s alphas compared to both commitment scales), we concluded that the U-MICS is suitable to assess identity dimensions among Zambian adolescents.

^3^The questionnaire GOALS also includes the domain of intimacy.However, this domain rather maps personal strivings toward romantic relationships. Thus, given the focus of our study, we selected affiliation-related goals that better match the identity domain of friendship and the assessment of *n* Affiliation in the present study.

^4^When inspecting internal consistencies of self-report scales, it was generally shown that the exclusion of single items was not an option to substantially enhance Cronbach alphas of scales.

^5^We also inspected correlations among indicators of well-being separately in both cultural samples. Life satisfaction, positive and negative affect, and general anxiety were significantly associated with each other (*p*s ≤ 0.001) in the German subsample. Similar relationships were found in the Zambian sample for components of subjective well-being (*p*s ≤ 0.001). Moreover, general anxiety significantly correlates with both negative affect (*p* ≤ 0.001) and life satisfaction (*p* = 0.008) but does not significantly relate to positive affect (*p* = 0.339). We additionally examined the EFA-based one-factor solution of measures of subjective well-being by multigroup CFA (unconstrained model: 28 data points and 24 unknown parameters to be estimated). The results show that the unconstrained model reasonably fit the data (e.g., χ^2^ = 51.13; df = 4; GFI = 0.935; RMSEA = 0.112); all items significantly loaded on the specified factor in both cultural samples (factor loadings |0.32| to |0.75|; critical ratios > |4.13|; *p*s < 0.001). Constraining factor loadings to be equal across cultural samples did not impair but rather slightly enhanced the model fit (∆GFI = 0.000; ∆RMSEA = −0.027).

^6^For brevity’s sake, a well-being index was generated from life satisfaction, positive and negative affect, and general anxiety. Their respective associations with identity dimensions in the total sample are presented in the following: Commitment is positively related to levels of self-reported positive affect (*r* = 0.20***), life satisfaction (*r* = 0.16***), and general anxiety (*r* = 0.07*) but unrelated to negative affect (*r* = 0.00). Reconsiderations of commitment shows positive associations with negative affect (*r* = 0.28***) and general anxiety (*r* = 0.19***) and negative relationships with positive affect (*r* = 0.13***) and life satisfaction (*r* = 0.21***). Finally, exploration in-depth positively relates to positive (*r* = 0.11***) and negative affect (*r* = 0.20***) and general anxiety (*r* = 0.26***) but shows no significant association with life satisfaction (*r* = −0.01).

^7^Additional analyses (model 7) were performed with identity dimensions in the domain *peers* as mediators in the proposed model. Although a mediating effect of both commitment-related dimensions could be verified, a significant moderation effect of *n* Affiliation was present in none of the analyses. Then, analyses were rerun with a combined index of identical identity dimensions in the domains *best friend* and *peers* (*r*s ranging from 0.57 to 0.63; *p*s < 0.001). The findings are very much in line with the results gained in analyses on *best friend* dimensions, although, not surprisingly, effects were weaker to some extent.
